# A serine-type protease activity of human lens βA3-crystallin is responsible for its autodegradation

**Published:** 2010-11-02

**Authors:** R. Gupta, J. Chen, O.P. Srivastava

**Affiliations:** 1 Department of Vision Sciences, University of Alabama at Birmingham, Birmingham, AL; 2 Cornell University, Ithaca, NY

## Abstract

**Purpose:**

The purpose of the study was to determine whether the autodegradation of human βA3-crystallin is due to its intrinsic protease activity.

**Methods:**

Recombinant His-tagged human βA3-crystallin was expressed in *E. coli* and purified by a Ni^+2^-affinity column chromatographic method. To determine protease activity, the purified crystallin was incubated for 24 h with either sodium deoxycholate, Triton X-100, or CHAPS {3-[(3-Cholamidopropyl) dimethylammonio]-1-propanesulfonate} and with benzoyl DL-arginine p-nitroanilide (BAPNA), a colorimetric protease substrate. The autodegradation of the crystallin at 0 h and 24 h on incubation at 37 °C with and without detergents (CHAPS/Triton X-100) was also determined by sodium dodecylsulfate-PAGE (SDS–PAGE) method. To examine whether the autodegradation of the crystallin was due to its protease activity, the crystallin was incubated with inhibitors of serine-, metallo- and cysteine-proteases. The binding of the intact βA3-crystallin and its autodegradation products to FFCK [5-carboxyfluorescenyl-1-phenylalaninyl-chloromethyl ketone], an analog of TPCK [1-Chloro-3-tosylamido-4-phenyl-2-butanone, a chymotrypsin-type serine protease inhibitor] was also determined by their incubation followed by SDS–PAGE and scanning for fluorescence using a Typhoon 9400^TM^ scanner.

**Results:**

βA3-crystallin protease activity showed activation in the presence of CHAPS but not in presence of Triton X-100. Upon incubation of βA3-crystallin for 24 h with CHAPS or sodium deoxycholate and BAPNA as a substrate, a time-dependent increase in the Arg-bond hydrolyzing activity was observed. SDS–PAGE analysis exhibited autodegradation products with M_r_ of 22, 27 and 30 kDa, which on partial NH_2_-terminal sequencing showed cleavage of Lys_17_-Met_18_, Gln_4_-Ala_5_ and Thr-Gly (in the NH_2_-terminal His-tag region) bonds, respectively. Almost no autodegradation of the βA3-crystallin occurred during its incubation alone or with CHAPS plus serine protease inhibitors (phenylmethylsulfonyl fluoride [PMSF], approtinin, and chymostatin). In contrast, the autodegradation occurred in the presence of metallo-protease inhibitors (EDTA and EGTA) and cysteine protease inhibitors (E-64, N-methylmaleimide and iodoacetamide). The βA3-crystallin also exhibited binding to FFCK, suggesting existence of a chymotrypsin-type active site in the βA3-crystallin protease.

**Conclusions:**

The results suggested that a serine-type protease activity of βA3-crystalllin was responsible for its autodegradation. The specific bonds cleaved during autodegradation (Gln_4_-Ala_5_ and Lys_17_-Met_18_), were localized in the NH_2_-terminal arm of βA3-crystallin.

## Introduction

The vertebrate lens structural proteins (crystallins) belong to two families, i.e., α-crystallin and the β-γ-crystallin superfamily. α-Crystallin is made of two primary gene products of αA- and αB-crystallin, the β-γ superfamily is constituted by four acidic (βA1-, βA2-, βA3-, and βA4-), three basic (βB1-, βB2-, and βB3-) β-crystallins, and six γ-crystallins (γA-, γB-, γC-, γD-, γE-, and γF) [1,2]. The high concentration of crystallins, their interactions and specific conformations provide the refractive power to the lens for focusing incoming light on to the retina. All crystallins are structural proteins except αA- and αB-crystallins, which are heat shock proteins with “chaperone” activity and belong to small heat shock protein family [3,4]. The expression of crystallins are both developmentally and spatially regulated [5], and their interactions lead to the transparency of the lens due to short-range order of the crystallin matrix [6]. Although β- and γ-crystallins have only structural properties [1,2,5], our results have shown that βA3-crystallin contains protease activity [7,8]. The βA3-crystallin protease hydrolyzed an Arg-bond containing substrate, but exists in an inactive state in the lens water soluble (WS) protein- [7], α-crystallin- and membrane-fractions [8], and is activated by detergents such as sodium deoxycholate [7,8]. We recently purified the βA3-crystallin-protease from the human lens α-crystallin fraction following its activation by a detergent. The purified enzyme exhibited proteolysis of αA- αB-, and γD-crystallins with specific cleavage of M_1_-G_2_, Q_54_-Y_55_, M_70_-G_71_, and Q_103_-M_104_ bonds in γD-crystallin [8]. This study also showed that a previously identified Arg-bond hydrolyzing 25 kDa- and membrane-proteases [9-11] are indeed identical to the βA3-crystallin protease.

Because the vertebrate lens contains several endopeptidases (i.e., a neutral protease [a multicatalytic proteosome] [12,13]; Calpain I and Calpain II [14]; a Lp82 calpain [15]; a Ca^+2^-dependent protease [16]; caspases-3 and −6 [17-19]; and matrix metalloproteases [MMP]-1, −2, −3, and −9 [20], and ubiquitin proteosome activity [21]), their wide substrate specificity made it difficult to study the βA3-crystallin-protease activity in a lens homogenate. The problem becomes more complicated because of very low protein turnover rate in the lens [22,23] that does not allow distinction of in vivo generated proteolytic products of one protease from other. This is in spite of a limited degradation of α-, β-, and γ-crystallins in the young human lenses, which increases with aging and cataractogenesis [24-28].

In view of lens very low protein turnover and the existence of several above described proteases, our previous results showing an association of protease activity with βA3-crystallin is intriguing. Because βA3-crystallin-protease exists in an inactive state in vivo, and following detergent-induced activation, it exhibited proteolysis of αA-, αB-, γC-, and γD-crystallins [8], apparently the crystallin enzyme activity must be tightly regulated in vivo. Presently, the nature of these intrinsic and/or extrinsic regulatory elements is unclear. Because α-crystallin has been shown to inhibit trypsin, elastase [29,30], and an endogenous lens protease [31], the potential role of α-crystallin as an inhibitor βA3-crystallin-protease is a possibility. This is partly evident by: (a) the detergent-induced activation of βA3-crystallin enzyme and its release from the α-crystallin fraction [8], and (b) our report showing that the motifs III plus IV and motifs II plus III of βA3-crystallin interact with αA- and αB-crystallins, respectively [32]. Because the activation of βA3-protease leads to truncation in its NH_2_-terminal arm [7], whether the truncation plays a role in activation of the enzyme is presently unknown. Previous reports show that the NH_2_-terminal truncation of βA3-crystallin occurs in vivo at an early age in both bovine and human lenses [33-35]. In human lenses, NH_2_-terminal truncation of 22 NH_2_-terminal residues in crystallin occurred early in life (i.e., 4 days-old) [34,35]. We have also reported an age-related progressive NH_2_-terminal truncation in human βA3-crystallin that produced a 4- to 18-kDa species with a prominent cleavage site at the E_39_-N_40_ bond [36]. These truncations might disrupt the stability of the βA3-crystallin because our results have shown that the NH_2_-terminal domain of βA3-crystallin is relatively more stable than the COOH-terminal domain and that βA3-crystallin without 21 or 22 NH_2_-terminal residues (or the entire NH_2_-terminal arm: residues number 1–30) is partially insoluble relative to WT protein [37].

To determine the importance of the truncation of residues in the NH_2_-terminal arm of the crystallin, the present study examined whether the truncation within the NH_2_-terminal arm of βA3-crystallin is due to its protease activity. Our results show that activation of the enzyme activity in the crystallin occurred on treatment with CHAPS, which was accompanied with autodegradation of the crystallin into two major species of 22 and 27 kDa with cleavage of Lys_17_-Met_18_ and Gln_4_-Ala_5_ bonds, respectively. Because the detergent-induced autodegradation of the crystallin could be prevented by serine protease inhibitors, the enzyme active site is a serine-type.

## Methods

### Materials

Unless indicated otherwise, all molecular biology grade chemicals used in this study were purchased from either Fisher (Atlanta, GA) or Sigma (St. Louis, MO).

### Purification of recombinant βA3-crystallin

The purification of recombinant βA3-crystallin was performed as described in our previous report [37]. Briefly, the βA3-crystallin was grown in Luria Broth media and expressed in *E. coli*, using 1 mM isopropyl β-D-1-thiogalactopyranoside (IPTG). The cells were harvested, lysed in a lysis buffer (25 mM Tris-HCl, pH 8.0; 50 mM NaCl; 50 mM glucose; 1mM EDTA (EDTA); 1% protease inhibitor cocktail; 1 mg/ml lysozyme), sonicated, and then centrifuged to separate the soluble fraction from the insoluble proteins. Next, the nickel-affinity column chromatography was used to purify the His-tagged βA3-crystallin. Briefly, following protein binding to a Ni^2+^-column (Invitrogen, Carlsbad, CA) in a native binding buffer (NBF; 50 mM phosphate, pH 8.0 containing 0.5 M NaCl), the non-specifically bound proteins were washed off with NBF buffer containing 20 mM imidazole. Next, the bound βA3-crystallin was eluted with NBF buffer containing 250 mM imidazole. The eluted fractions were analyzed by SDS–PAGE [38] using a 15% polyacrylamide gel to confirm the presence of a 31 kDa βA3-crystallin protein band. The fractions containing βA3-crystallin were then pooled, concentrated by lyophilization, dialyzed against 50 mM phosphate buffer, pH 7.5 at 4 °C for 48 h, and stored at −20 °C until used.

### Assay of βA3-crystallin proteinase activity

The enzymatic activity was assayed colorimetrically with benzoyl-DL-arginine-p-nitroanilide (BAPNA) as a substrate [39,40].

### Activation of protease activity and autodegradation of βA3-crystallin following detergent treatment

Either a non-ionic (1%, Triton X-100), a zwitterionic (0.2%, CHAPS) or an anionic (sodium deoxycholate, 0.5%) detergent was used to activate the protease activity in the purified recombinant βA3-crystallin preparation. In some experiments, the βA3-crystallin (20 μg), a detergent, and BAPNA were mixed before incubation at 37 °C for 24 h, while in other experiments, the crystallin and a detergent were incubated together for 1 h and then BAPNA was added with the incubation mixture at 37 °C and monitored for protease activity for up to 24 h. BAPNA released p-nitroaniline (yellow) on its hydrolysis by βA3-protease, which was monitored at 405 nm. To determine the autodegradation of the crystallin in the presence or absence of CHAPS or Triton X-100, their mixtures were incubated for 24 h and then analyzed by SDS–PAGE using 15% polyacrylamide gels. The effect of inhibitors of serine-, metallo- and cysteine-proteases on the autodegradation of βA3-crystalllin in the presence of CHAPS was determined by incubation of a mixture of the crystallin, CHAPS and protease inhibitor together for 24 h and then examination by SDS–PAGE.

### Binding of βA3-crystallin-proteinase with FLISP^TM^ reagent

FLISP (Fluorescent Labeled Inhibitors of Serine Proteases) reagents from Immunochemistry Technology (Bloomington, MN) are fluorescent probes. One such probe is FFCK (5(6)-carboxyfluoroscenyl-l-phenylalaninyl-chloromethyl ketone), which is an analog of TPCK (1-Chloro-3-tosylamido-4-phenyl-2-butanone), a well known chymotrypsin-type serine protease inhibitor. FFCK exhibits substrate specificity for phenylalanine, acts as a pseudosubstrate for chymotrypsin-like serine proteases, covalently binds to activated proteases, and it can be used to measure increases in the reactivity of catalytic sites as proteases are activated. In our experiment, the binding of CHAPS-treated βA3-crystallin and a preparation containing βA3-crystallin autodegradation products were incubated with FFCK (25 μM, final concentration) at 37 °C for 30 min, and then the binding was determined following SDS–PAGE and scanning the gel for fluorescence using a Typhoon 9400^TM^ scanner (GE Healthcare, Piscataway, NJ) with excitation set at 488 nm and emission at 520 nm.

### Miscellaneous methods

Protein concentration was determined by using Pierce BCA Protein Assay Reagent (bicinchorininic acid; Thermo Scientific, Wilmington, DE). Determination of cleavage sites in βA3-crystallin following autodegradation was performed by NH_2_-terminal sequencing using Edman degradation method. Briefly, the truncated species were separated by SDS–PAGE using a 15% polyacrylamide gel, and next the proteins from the gel were electrophoretically transferred to a polyvinylidene fluoride (PVDF) membrane (BioRad, Hercules, CA) by the method of Towbin et al. [41]. The transferred proteins were stained with Coomassie blue and destained before sequencing. The desired protein bands were NH_2_-terminally sequenced at the Protein Facility of Iowa State University-Office of Biotechnology (Ames, IA). The mass spectrometric analysis of desired samples was performed by quadruple ion trap (Q-TRAP) mass spectrometry at the Mass Spectrometric Core Facility of the University of Alabama at Birmingham (Birmingham, AL). The desired protein bands were excised and subjected to in-gel trypsinization before mass spectrometric analysis. The MASCOT (Matrix Science) program was used to search for human lens and *E. coli* proteins in samples. The database used for the searches was the UniProt database.

## Results

### Purification of recombinant βA3-crystallin, and its detergent-induced activation of protease activity

**Figure 1:**
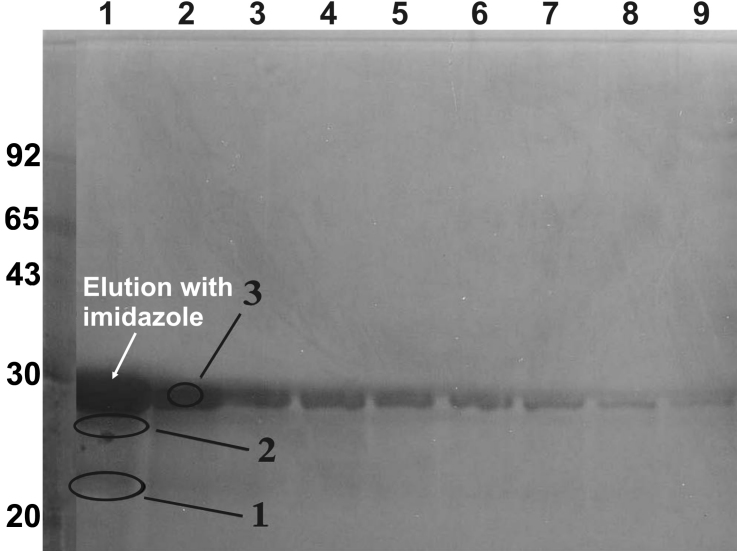
Purification of recombinant βA3-crystallin by the nickel-affinity column chromatographic method. During purification of βA3-crystallin from the soluble protein fraction of the *E. coli* cell lysate, the bound proteins were eluted using 250 mM imidazole, and each fraction was analyzed using a 15% polyacrylamide gel using the SDS–PAGE method. Fractions (lanes 1 to 9) containing the single major protein band of βA3-crystallin were pooled, dialyzed, and used for further experiments. Circled bands 1, 2, and 3 were excised from gels, trypsin-digested and analyzed by quadruple ion trap (Q-TRAP) mass spectrometric method. The analysis identified these circled species (1, 2, and 3) as βA3-crystallin suggesting that the parent crystallin was partially degraded to produce two minor crystallin fragments during purification.

The purification of recombinant βA3-crystallin was performed using a Ni^+2^-affinity column chromatographic method as previously described [37]. Upon elution of βA3-crystallin with 250 mM imidazole, highly purified preparation of the crystallin was recovered as shown in lanes 1 to 9 of Figure 1. Because of the His-tag, βA3-crystallin showed a M_r_ of ~31 kDa (identified as major band number 3 in Figure 1) instead of a M_r_ of 27 kDa for a non-His tagged native βA3-crystallin. Further, two minor lower molecular weight species (identified a bands 1 and 2 in Figure 1) were also observed. The band numbers 1, 2, and 3 were identified as βA3-crystallin by the Q-TRAP mass spectrometric method, suggesting partial degradation of βA3-crystallin during purification. The QTRAP data were analyzed against *E. coli* and human databases, which showed an absence of any *E.coli* proteases in the purified βA3-crystallin preparation. The fractions containing the highly purified intact βA3-crystalllin were pooled, concentrated by lyophilization, dialyzed against 0.05 M phosphate buffer, pH 7.5, and stored at −20 °C until used. The purified βA3-crystallin fraction showed mainly the intact crystallin but minor quantities of two truncated species of 22 and 27 kDa were also present, which could be attributed to basal level of truncation of the intact crystallin due to its protease activity.

The protease activity in the purified βA3-crystallin was determined with BAPNA as the substrate by two methods. The first method was similar to our previously described method [7], where the crystallin was treated with either sodium deoxycholate (0.5%, w/v, final concentration) or CHAPS (0.2%, w/v, final concentration), and immediately fractionated by HPLC using a TSK G-4000 PW_XL_ column (Tosoh Bioscience, Montogomeryville, PA). Next, the eluted fractions were assayed with BAPNA as a substrate at 37 °C for 24 h. In the second method, the crystallin was incubated with CHAPS/Triton X-100 and BAPNA at 37 °C, and protease activity was monitored at 405 nm with BAPNA or by SDS–PAGE for crystallin truncation at desired intervals up to 24 h.

**Figure 2:**
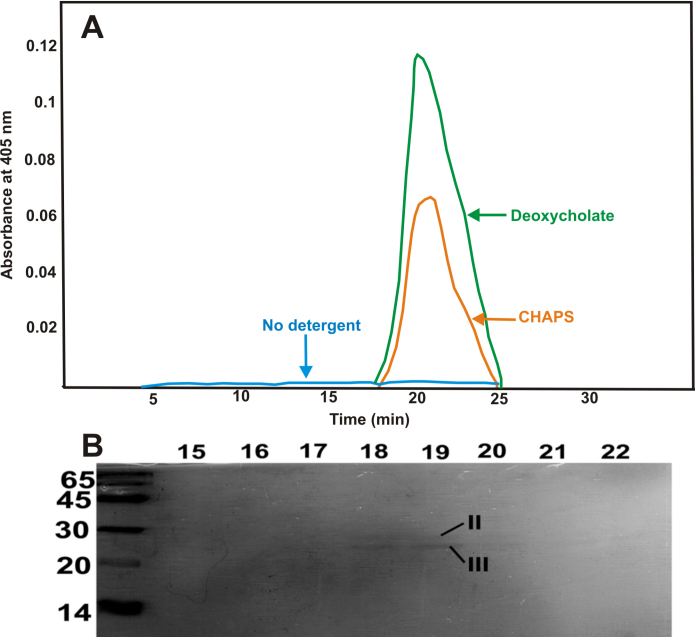
Activation of protease activity in recombinant βA3-crystallin following treatment with sodium deoxycholate or CHAPS. **A**: The βA3-crystallin preparations (200 μg), treated with sodium deoxycholate or CHAPS, showed protease activity after HPLC fractionation whereas no activity was observed in the detergent-untreated crystallin fraction. **B**: On SDS–PAGE analysis of the column fractions containing protease activity, two species of 22 and 27 kDa were observed with an absence of the parent 31 kDa βA3-crystallin species. Results suggested a degradation of βA3-crystallin upon activation of its protease activity.

**Figure 3:**
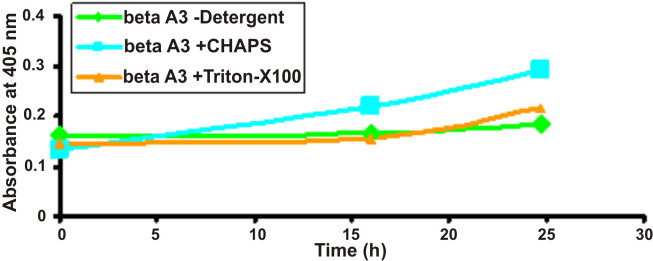
Assay of protease activity in the βA3-crystallin following treatment with CHAPS or Triton X-100. Following treatment of βA3-crystallin preparation (50 μg) with either CHAPS or Triton X-100 and incubation with BAPNA as a substrate, only the CHAPS-treated crystallin exhibited protease activity. The protease activity increased during incubation for 24 h at 37 °C as indicated by the release of p-nitroaniline (yellow) from BAPNA, which was monitored at 405 nm.

As shown in Figure 2A, the βA3-crystallin preparations treated with either sodium deoxycholate or CHAPS showed protease activity after HPLC fractionation whereas no activity was observed in the detergent-untreated fraction. Further, the SDS–PAGE analysis of the column fractions containing protease activity showed 22 and 27 kDa bands instead of a parent 31 kDa βA3-crystallin band (Figure 2B). This suggested that truncation of βA3-crystallin occurred following CHAPS-induced activation of protease activity in the crystallin. During the assay of protease activity in the crystallin by the second method of incubation with BAPNA and with and without CHAPS or Triton X-100, only the CHAPS-treated crystallin preparation exhibited a time-dependent increase in protease activity at 37 °C during 24 h of incubation (Figure 3).

### Autodegradation of βA3-crystallin with cleavage of specific bonds upon treatment with CHAPS

**Figure 4:**
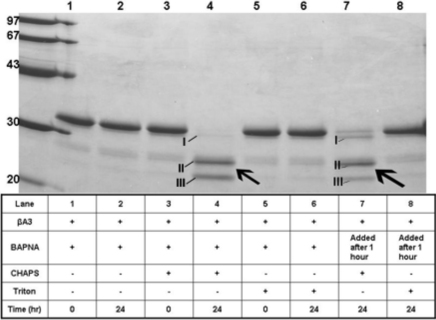
Signature truncation of βA3-crystallin upon incubation with CHAPS. Upon incubation of βA3-crystallin (50 μg) with CHAPS for 24 h at 37 °C in the presence of BAPNA, an increase in the protease activity was accompanied with autodegradation of the crystallin. Lane 1: 0 h incubation and lane 2: 24 h incubation of the crystallin alone without CHAPS; lanes 3 [0 h] and 4 [24 h]) incubation with CHAPS; lanes 5 (0 h) and 6 (24 h) incubation with Triton X-100; lane 7: incubation with CHAPS plus BAPNA, and lane 8: incubation with Triton X-100 and BAPNA. Note the autodegradation of the crystallin to three truncated species (identified as I, II, and III) in the presence of CHAPS but not in the presence of Triton X-100.

To understand the nature of the βA3-crystallin-protease, non-ionic (Triton X-100) and zwitterionic (CHAPS) detergents were used, and the protease activity was determined as described above using BAPNA as a substrate. Upon incubation of βA3-crystalllin with CHAPS at 37 °C for 24 h, an increase in the protease activity was accompanied with autodegradation of the crystallin (Figure 4). Lanes 1 and 2 in Figure 4 show that on incubation of the crystallin alone without detergent at 37 °C for 0 and 24 h, respectively, resulted in almost no autodegradation. In contrast, autodegradation of the crystallin after 24 h of incubation with CHAPS occurred (Figure 4; lanes 3 [0 h] and 4 [24 h]). The autodegrdation resulted in the appearance of three major truncated species, namely I, II, and III, which exhibited M_r_ of 30, 27, and 22 kDa, respectively. No autodegradation of the crystallin occurred on incubation with Triton X-100 at 37 °C for 24 h as shown in lanes 5 (0 h) and 6 (24 h) in Figure 4. In lanes 7 and 8, BAPNA was added after 1 h of incubation of βA3 with CHAPS or Triton X-100. The autodegradation pattern was similar with the appearance of species I, II and III in the presence of CHAPS plus BAPNA (Figure 4, lane 7), suggesting that the presence of BAPNA did not affect the autodegradation process. No autodegradation of crystallin occurred in the presence of Triton X-100 and BAPNA on incubation at 37 °C for 24 h (Figure 4, lane 8). Because no protease activity in the crystallin was observed on incubation of the crystallin with Triton X-100 (Figure 3), the lack of autodegradation in the crystallin in the presence of Triton X-100 suggest that it must be associated with the protease activity. Aliquot from each reaction mixture was monitored at 405 nm and an increase in protease activity after incubation of 24 h compared to 0 h was observed (data not shown).

Next, it was determined whether CHAPS-induced autodegradation of crystallin could generate more than three truncated species (i.e., I, II, and III, Figure 4) upon increased incubation time at 37 °C. On incubation for up to 76 h of the crystallin with the CHAPS, again mainly three truncated species (I, II, and III) were observed whereas no autodegradation occurred in the absence of the CHAPS (results not shown). The data suggested that even after incubation upto 76 h, the CHAPS-induced autodegradation of βA3 resulted in mainly three major species shown as I, II, and III.

The cleavage sites in βA3-crystallin to generate truncated species I, II, and III were determined by partial NH_2_-terminal sequencing by the Edman Degradation method. Fragment I showed a sequence of M-G-G-Q-Q, which suggested that the cleavage of Q-M bond in the His-tag region resulting in the production of truncated species I. Fragments II and III showed partial NH_2_-terminal sequences of A-E-Q-Q-E and M-A-Q-T-N, respectively, suggesting that the truncated species II and III were generated following cleavage of Q_4_-A_5_ and K_17_-M_18_ bonds in βA3-crystallin, respectively. Taken together, the above results show that His-tagged βA3-crystallin on autodegradation produced three signature truncated species of 30, 27, and 22 kDa species with cleavage sites at Q-M bonds in the His-tag region, Q_4_-A_5_ and K_17_-M_18_ bonds in the NH_2_-terminal arm of βA3-crystallin, respectively.

### Inhibition of autodegrdation of βA3-crystalllin by protease inhibitors

**Figure 5:**
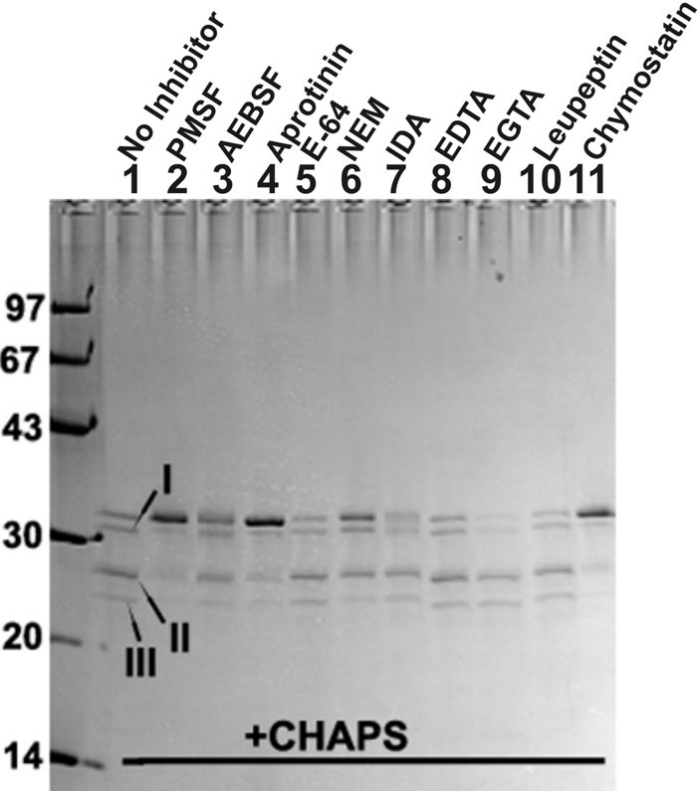
Inhibition of autodegrdation of βA3-crystalllin by protease inhibitors in the presence of CHAPS. The criterion for inhibition in the study was whether an inhibitor was able to prevent signature autodegradation of βA3-crystallin (50 μg, used with each inhibitor) into the three truncated specific I, II, and III. The inhibitors used in the study are identified at the top of the gel. Only the inhibitors of serine-type proteases such as phenylmethyl sulfonyl fluoride (PMSF, 2 mM, lane 2), aprotinin (25 μg/ml, lane 4) and chymostatin (lane 11) inhibited autodegradation of the crystallin, whereas 4(2-aminoehtyl)-benzene sulfonyl fluoride (2 mM, lane 3) showed partial inhibition of autodegradation. In contrast, the cysteine-protease inhibitors (E-64, 100 μM [lane 5], N-ethylmaleimide, 5 mM [lane 6] and iodoacetamide, 5 mM [lane 7], and metallo-proteinase inhibitors (EDTA, 5 mM [EDTA, lane 8] and ethylene glycotetraacetic acid, 5 mM [EGTA, lane 9] did not stop autodegradation of the crystallin.

The autodegradation of βA3-crystallin in the presence of various inhibitors of proteases was determined (Figure 5). The criterion for inhibition in the study was whether an inhibitor was able to prevent signature autodegrdation of the crystallin into the three truncated specific I, II, and III. Because the experiments of Figure 4 suggested that the autodegrdation was dependent on activation of protease activity, it was assumed that the inhibitors that prevented autodegradation also inhibited activation of the βA3 protease activity. As shown in Figure 5, only the inhibitors of serine-type proteases such as phenylmethylsulfonyl fluoride (PMSF, 2 mM, lane 2), aprotinin (25 μg/ml, lane 4) and chymostatin (1 mM, lane 11) inhibited autodegradation of the crystallin, whereas 4(2-aminoethyl) benzene sulfonyl fluoride (2 mM, lane 3) was only partially inhibitory. In contrast, the cysteine-protease inhibitors (E-64 [Figure 5, lane 5], N-ethylmaleimide [Figure 5, lane 6] and iodoacetamide [Figure 5, lane 7], and metallo-proteinase inhibitors (EDTA [EDTA, Figure 5, lane 8] and ethylene glycol tetraacetic acid, [EGTA, Figure 5, lane 9] did not stop degradation of the crystallin. The data suggested that the CHAPS-induced degradation of the crystallin was due to serine-type protease activity associated with βA3-crystallin. Next, a water soluble serine protease inhibitor, AEBSF (4-[2-aminoethyl] benzenesulfonyl fluoride) was used at an increasing concentrations to examine if aurodegradation of the crystallin could be prevented. On incubation of the crystallin with CHAPS and varied concentration of AEBSF inhibitor between 2 to 8 mM, the autodegradation of the crystallin was almost prevented in a dose-dependent manner (results not shown). However, even at the highest level of the inhibitor, minor quantities of two truncated species of 22 and 27 kDa species were also present, which could be attributed to basal level of truncation of the intact crystallin due to autolytic activity of βA3-crystallin-protease.

### Binding of βA3-proteinase with FLISP^TM^ reagent

**Figure 6:**
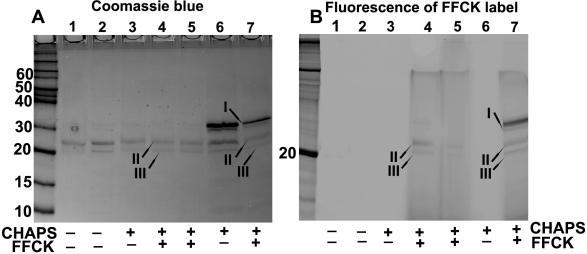
SDS–PAGE analysis and fluorescence determination to examine binding of FFCK to untruncated βA3-crystallin and its two major truncated products. Lanes 4 and 5 represents βA3-crystallin truncated species, which were recovered following treatment of βA3-crystallin with CHAPS, and were labeled with FFCK. The lanes 6 and 7 represented βA3-crystallin treated with CHAPS but complete degradation of intact crystallin to 22 and 27 kDa species was not allowed to determine if all three species (intact, 22, and 27 kDa species) show labeling with FFCK. The untruncated βA3-crystallin (Coomassie blue stained in lane 7 of **A**) and its two truncated species of 22 and 27 kDa (Coomassie blue-stained bands in lane 4 and 7 in **A**) showed binding to FFCK (25 μM, final concentration) as represented by fluorescent bands (lane 4 and 7 in **B**). The results suggest that because of FFCK binding, the protease active site is present in both untruncated βA3-crystallin and its major truncated species II and III.

As stated above, the FLISP reagents covalently binds to activated protease and an increasing binding represent increasing number of active site becoming available as a protease activates. Because the CHAPS-induced degradation of βA3-crystallin could be inhibited by chymostatin, the protease activity could be chymotrypsin type (Figure 5, lane 11). To determine this, a probe FFCK (5 [6]-carboxyfluoroscenyl-l-phenylalaninyl-chloromethyl ketone), which is an analog of TPCK (1-Chloro-3-tosylamido-4-phenyl-2-butanone, a well known chymotrypsin-type serine protease inhibitor) was used. The purpose of the experiment was to test the binding of FFCK to both intact and truncated forms of βA3-crystallin. Therefore, for the FFCK-labeling, we selectively used those preparations which either contained only the two major degradation products βA3-crystallin or intact βA3-crystallin plus the two degradation products. The truncated forms of βA3-crystallin shown in Figure 6, lanes 1–5 were generated by pretreatment of the crystallin with CHAPS, while CHAPS was only added to the βA3-crystallin in lanes 6 and 7 (Figure 6) during the 30 min incubation with FFCK. Lanes 4 and 5 (Figure 6) represents βA3-crystallin truncated species, which were recovered following treatment with CHAPS, and were labeled with FAM. The lanes 6 and 7 represented βA3-crystallin treated with CHAPS but complete degradation of intact crystallin to 22 and 27 kDa species was not allowed to determine if all three species (intact, 22 and 27 kDa species) show labeling with FFCK. As shown in Figure 6, the intact βA3-crystallin (Coomassie blue stained in lane 7 of Figure 6A) and its two truncated species of 22 and 27 kDa (Coomassie blue stained bands in lanes 4 and 7 in Figure 6A) showed binding to FFCK as represented by fluorescent bands (lanes 4 and 7 in Figure 6B). Based on FFCK binding, the results suggest that the protease active site is present in both intact βA3-crystallin and its two truncated species II and III.

## Discussion

In this study, the His-tagged recombinant βA3-crystallin was expressed in *E.coli,* and used because of its easy one step purification by Ni^+2^-affinity column chromatographic method. This fulfilled one of the requirements of the present study to carry out desired experiments with a highly purified βA3-crystalin preparation. The purified βA3-crystallin showed a single band during both SDS–PAGE and two-dimensional (2-D) gel electrophoresis, and its identity was confirmed by Q-TRAP mass spectrometric method (results not shown). As stated above, we determined whether any *E.coli* protease was recovered as a contaminant during purification of βA3-crystalllin with either intact crystallin or its two 22 and 27 kDa truncated species (Figure 1, protein bands 1, 2, and 3), the Q-TRAP mass spectrometric results were analyzed against *E.coli* and human databases. The analysis revealed an absence of any protease from *E.coli* with the human βA3-crystallin. This highly purified recombinant βA3-crystallin preparation was used in the present study.

The major findings of the study were: (1) the activation of protease activity in βA3-crystallin occurred by treatment with CHAPS (a zwitterion detergent) or sodium deoxycholate (an anionic detergent) but not with Triton X-100 (a non-ionic detergent). (2) The activation of the protease activity in βA3-crystallin led to specific signature truncations in the crystallin with appearance of three truncated species of 22, 27, and 30 kDa, which were named as species I, II, and III, respectively. (3) The partial NH_2_-terminal sequence of species I (sequence: M-G-G-Q-Q), species II (sequence A-E-Q-Q-E) and species III (M-A-Q-T-N) suggested that the crystallin protease activity autolytically cleaved Q-M bonds in the His-Tag region, and Q_4_-A_5_ and K_17_-M_18_ bonds in the NH_2_-terminal arm. (4) Because the signature cleavages of βA3-crystallin to produce species I, II, and III could be prevented by serine-type protease inhibitors but not by cysteine- or metallo-protease inhibitors, the autolytic cleavages were due to a serine-type protease activity of βA3-crystallin. (5) FFCK (5 [6]-carboxyfluoroscenyl-l-phenylalaninyl-chloromethyl ketone), an analog of TPCK ([1-Chloro-3-tosylamido-4-phenyl-2-butanone] and a well known inhibitor of chymotrypsin-type serine proteases, showed binding with native βA3-crystallin and its truncated species, suggesting that the active site of the enzyme to be chymotrypsin-type, and is intact in the native βA3-crystallin and its major two truncated species.

An association of protease activity with βA3-crystallin is intriguing in view of the close homology between βA3-crystallin and other β- and γ-crystallin species. The βB1-, βB2-, and βA3-crystallins are composed of two significantly homologous domains that are connected by a short linker sequence. Each domain exhibits two “Greek Key” motifs [42-45]. The significant sequence homology between βB2- and βA3-crystallins suggests they share similar structures, but the biophysical evidence indicates that βA3-crystallin aggregates to a relatively higher molecular-weight form. Our results suggest that “activation” of the protease activity in βA3-crystallin might be due to deoxycholate treatment, which removes monomeric or dimeric βA3-crystallin molecules from the higher aggregation states [7]. The α-crystallin has also been shown to dissociate from higher aggregated states by sodium deoxycholate [46]. Additionally, the truncation of ~20 NH_2_-terminal residues and the secondary and tertiary structural changes in βA3-crystallin might also play a role in its acquiring protease activity [8].

As stated above, the potential effect of NH_2_-terminal truncation on βA3-crystallin is unknown, but it occurs at an early age in both bovine and human lenses [33-35]. In human lenses, NH_2_-terminal truncation of 22 NH_2_-terminal residues in βA3-crystallin occurs very early in life (i.e., 4 day-old) [35]. Similarly, the truncated βA3-crystallin species, missing either 11 or 22 NH_2_-terminal residues, were observed in the fetal bovine lens, and the latter became a major βA3-crystallin species in the adult lens [33,34]. Our previous report has shown that an age-related progressive NH_2_-terminal truncation in human βA3-crystallin produced a 4- to 18-kDa species with a prominent cleavage site at the E_39_-N_40_ bond [36]. The reason for these specific truncations and their effects on βA3-crystallin remains unknown. Our biophysical studies have demonstrated that truncations of 21 or 22 NH_2_-terminal residues or entire NH_2_-terminal extension (residue number 1–30) partially affect the solubility of the crystallin [37]. Previously, we have speculated that the activation of protease activity in βA3-crystallin might be associated with truncation of 22 NH_2_-terminal residues [7], and therefore this region could play a major role in regulation of the crystallin enzyme activity. Further, we observed that the purified recombinant βA3-crystallin showed partial degradation on storage at 4 °C or freezing and thawing, which could be due to its protease activity. While bovine lens m-calpain was implicated in the proteolysis of βA3-crystallin to produce a species missing 11 NH_2_-terminal residues [33], no lens protease has yet been identified to produce a βA3-crystallin species missing 22 NH_2_-terminal residues in either bovine or human lenses. This yet unknown lens protease cleaved N-P bond and specifically recognized N-P-X-P region in βA3-crystallin of both bovine and human lenses [35]. Our present study shows that the protease activity of βA3-crystallin is responsible for the cleavage Q_4_-A_5_ bond to produce βA3 species lacking four NH_2_-terminal residues but not specific for cleavage of P-X-P bond to produce the βA3-crystallin species missing 22 NH_2_-terminal residues. Therefore, a potential in vivo role of βA3-crystallin protease in the maturation process of βA3-crystalllin during normal lens development could not be implicated. The significance of the cleavage of NH_2_-terminal arm of βA3-crystallin during developmental process in vivo is presently unclear and is under investigation.

The cleavage of the NH_2_-terminal arm of other β-crystallins might be of significant because during selenite cataract in young rodents, an accelerated loss of NH_2_-terminal extensions in β-crystallins has been shown to lead to a rapid protein insolubilization and lens opacity [47]. Similarly, βA3-crystallin without NH_2_-terminal arm increased its susceptibility to aggregation on UV irradiation suggesting such loss in the maturing lens might lead to age-related cataracts [48].

The question whether in vitro detergent-induced activation of protease activity in βA3-crystallin is relevant to in vivo situation in the lens can presently be answered based on our preliminary results as the molecular mechanism of activation βA3-crystalllin protease remains unknown. Our results suggest that apparently the regulation of protease activity in βA3-crystalin occurs intrinsically by its NH_2_-terminal arm, and extrinsically by the αA- and αB-crystallins as a protease inhibitor. Because the truncation in the NH_2_-terminal arm leads to appearance of protease activity in βA3-crystallin, the former might be involved in the regulation of the protease activity. The potential role of α-crystallin as an inhibitor of βA3-crystallin protease was suggested by the presence of the enzyme in an inactive state in the α-crystallin fraction. The detergent treatment induced enzyme activation and its dissociation from the α-crystallin fraction, suggesting an inhibition of the enzyme by α-crystallin via hydrophobic interactions [8]. Such an interaction was supported by our report that identified the interacting motifs of βA3-crystallin with αA- and αB-crystallins [32]. The βA3-crystallin protease also existed in an inactive state in the lens membrane fraction, and could be activated with a detergent [8]. Together, these results suggest that apparently in vivo, βA3-crystallin protease exists in a hydrophobic environment, possibly with α-crystallin and membranes. Once the crystallin is dissociated from such an environment by a detergent in vitro, its active site is exposed due to conformation changes. In summary, the activation of the protease activity by CHAPS in our study provides a clue to how the protease activity might be activating in vivo.

The presence of the protease active site in untruncated βA3-crystallin was further supported by the FFCK binding as shown in Figure 6. It is clear from our studies that the activated protease activity leads to autolytic degradation of the crystallin only in the presence of CHAPS (a zwitterion) but not in presence of Triton X-100 (non-ionic). Although, both of these detergents bind to the hydrophobic patches of the protein, the differential response to non-ionic and zwitterionic detergent on βA3-crystallin protease activation may reflect intrinsic changes in the protein conformation resulting in exposure of the enzymatic active site. In this regard, the potential role of the cleavages at Q_4_-A_5_ and K_17_-M_18_ bonds in activation of the crystallin protease activity or in stabilizing the crystallin structure can not be ruled out.

A varied functional role of the NH_2_-terminal arm of βA3-crystallin has been suggested [49-52]. Our previous report has shown that the NH_2_-terminal domain of βA3-crystallin is relatively more stable than the COOH-terminal domain, and that compared to WT protein, βA3-crystallin without 21 or 22 NH_2_-terminal residues (or the entire NH_2_-terminal arm [residue number 1–30]) is partially insoluble [37]. Upon removal of 30 residues of the NH_2_-terminal extension of mouse βA3/A1-crystallin, its dimer formation was affected [50]. A ^1^H NMR study following deletion of the first 22 amino acids of the NH_2_-terminal extension showed that the deleted segment was not involved in oligomerization [49]. The loss of part of the NH_2_-terminal arm had no effect of the stability of βA3-crystallin; instead, the stability of the protein was slightly increased [49,52], which was also seen in our previous report [37]. Another study further showed that the NH_2_-terminal arm of βB2-crystallin retained its flexibility in βH- and βL-complexes, but the arm of βA3/A1-crystallin was constrained [51]. Further, the stability of NH_2_- and COOH-terminally cleaved rat βB2-crystallin was found to be lower than that of the full-length protein [49]. In summary, the above studies do not clearly define functional role of the NH_2_-terminal arm, and therefore, it is significant to determine what role the βA3-crystallin-protease plays in its own truncation of NH_2_-terminal arm and those of other β-crystallins. These studies are presently being conducted.
